# Sirt2‐BubR1 acetylation pathway mediates the effects of advanced maternal age on oocyte quality

**DOI:** 10.1111/acel.12698

**Published:** 2017-10-25

**Authors:** Danhong Qiu, Xiaojing Hou, Longsen Han, Xiaoyan Li, Juan Ge, Qiang Wang

**Affiliations:** ^1^ State Key Laboratory of Reproductive Medicine Nanjing Medical University Nanjing China; ^2^ College of Animal Science & Technology Nanjing Agricultural University Nanjing China

**Keywords:** aging, aneuploidy, meiosis, oocyte, sirtuin

## Abstract

The level of Sirt2 protein is reduced in oocytes from aged mice, while exogenous expression of Sirt2 could ameliorate the maternal age‐associated meiotic defects. To date, the underlying mechanism remains unclear. Here, we confirmed that specific depletion of Sirt2 disrupts maturational progression and spindle/chromosome organization in mouse oocytes, with compromised kinetochore–microtubule attachments. Candidate screening revealed that acetylation state of lysine 243 on BubR1 (BubR1‐K243, an integral part of the spindle assembly checkpoint complex) functions during oocyte meiosis, and acetylation‐mimetic mutant BubR1‐K243Q results in the very similar phenotypes as Sirt2‐knockdown oocytes. Furthermore, we found that nonacetylatable‐mimetic mutant BubR1‐K243R partly prevents the meiotic deficits in oocytes depleted of Sirt2. Importantly, BubR1‐K243R overexpression in oocytes derived from aged mice markedly suppresses spindle/chromosome anomalies and thereupon lowers the incidence of aneuploid eggs. In sum, our data suggest that Sirt2‐dependent BubR1 deacetylation involves in the regulation of meiotic apparatus in normal oocytes and mediates the effects of advanced maternal age on oocyte quality.

## 1 INTRODUCTION

In mammals, oocytes supply the embryo with the maternal genetic materials and the majority of organelles, membranes, and cytoplasmic components needed for development. Before acquiring the capabilities that are required for fertilization and embryogenesis, oocytes must go through a prolonged and complicated developmental process (Li & Albertini, 2013). Upon completion of early prophase I in which S‐phase and recombination are taking place in the fetal ovary, oocytes remain arrested in dictyotene stage with largely decondensed chromatin within the nucleus, termed “germinal vesicle” (germinal vesicle stage, GV) within primordial follicles up to decades in the human. Following luteinizing hormone (LH) stimulation in the adult, postpubertal female, fully grown oocytes enter meiosis I, a reductional division (Neal & Baker, 1975). At metaphase I (MI) stage, microtubules organize into the specialized barrel‐shaped bipolar spindle, with all chromosomes aligned at the equatorial plate. Anaphase I usually occurs when all chromosome bivalents have established stable kinetochore–microtubule (K‐MT) interactions. Then, oocytes enter directly into meiosis II without an intervening S‐phase and arrest at metaphase II (MII) stage waiting for fertilization (Solc, Schultz, & Motlik, 2010; Watanabe, 2012). Unfortunately, the meiotic divisions in oocytes are highly error prone (Fragouli et al., 2011; Kuliev, Zlatopolsky, Kirillova, Spivakova, & Cieslak Janzen, 2011). Oocytes with the wrong number of chromosomes give rise to aneuploid embryos when fertilized. The majority of aneuploid embryos are nonviable and spontaneously aborted. The frequency of meiotic defects and aneuploidy in oocytes is strongly associated with maternal age and increases exponentially in the decade preceding the menopause (MacLennan, Crichton, Playfoot, & Adams, 2015; Nagaoka, Hassold, & Hunt, 2012).

Sirtuins constitute an evolutionarily conserved family of NAD^+^‐dependent deacetylases. They have been implicated in diverse biological events, such as aging, energy control, circadian clocks, and mitochondrial biogenesis (Baur, Ungvari, Minor, Le Couteur, & de Cabo, 2012; Preyat & Leo, 2013; Wood et al., 2004). Of them, Sirt2 is associated with mitotic structures, beginning with the centrosome in mitotic prophase, the spindle in metaphase, and the midbody during cytokinesis, presumably to ensure normal cell division (North & Verdin, 2007). Consistent with this finding, we report that specific depletion of Sirt2 in mouse oocytes results in spindle defects and chromosome disorganization. Notably, lower Sirt2 protein level was detected in meiotically dividing oocytes from aged mice, and maternal age‐associated meiotic defects can be ameliorated through overexpression of Sirt2 (Zhang et al., 2014). Although histone H4K16 and α‐tubulin have been suggested to be the potential targets of Sirt2 in oocytes, the underlying mechanism connecting Sirt2 insufficiency and age dependent deficits remains elusive.

BubR1 (budding uninhibited by benzimidazole‐related 1), also called BUB1B in human, is a core component of the spindle assembly checkpoint (SAC) system (Sudakin, Chan, & Yen, 2001). Through SAC signal activation, BubR1 participates in proper chromosome segregation during mitosis (Baker et al., 2013; Lampson & Kapoor, 2005). BubR1, is required at several key steps in oocyte meiosis, and more specifically, for SAC activity, the timing of the first meiotic division, and the stable attachment of chromosomes to the spindle (Brunet, Pahlavan, Taylor, & Maro, 2003; Wei et al., 2010). In oocytes, BubR1 is required for the establishment of robust K‐MT attachments in a meiosis‐specific manner (Wei et al., 2010). It is worth noting that a marked reduction of BubR1 levels was detected in aged oocytes from both human and mice (Baker et al., 2004; Riris, Webster, & Homer, 2014). Recently, emerging evidence indicates the connection between Sirt2 and BubR1. In specific, Sirt2 was shown to be able to modulate BubR1 stability in vitro and in vivo through deacetylation (North et al., 2014; Suematsu et al., 2014). Moreover, numerous studies demonstrated that deacetylation status of BubR1 is essential for maintaining the mitotic structure integrity and the control of cell cycle (Choi et al., 2009; Park et al., 2013; Suematsu et al., 2014).

Given all these findings, we asked whether Sirt2‐controlled BubR1 acetylation is a critical mechanism determining oocyte quality, particularly mediating the meiotic defects induced by maternal age. In the present study, by employing the acetylation‐ and deacetylation‐mimetic mutants, we explored the function of BubR1 acetylation in meiotic oocytes and its relationship with the defective phenotypes of aged oocytes.

## 2 RESULTS

### 2.1 Sirt2 knockdown disturbs meiotic apparatus in mouse oocytes

**Figure 1:**
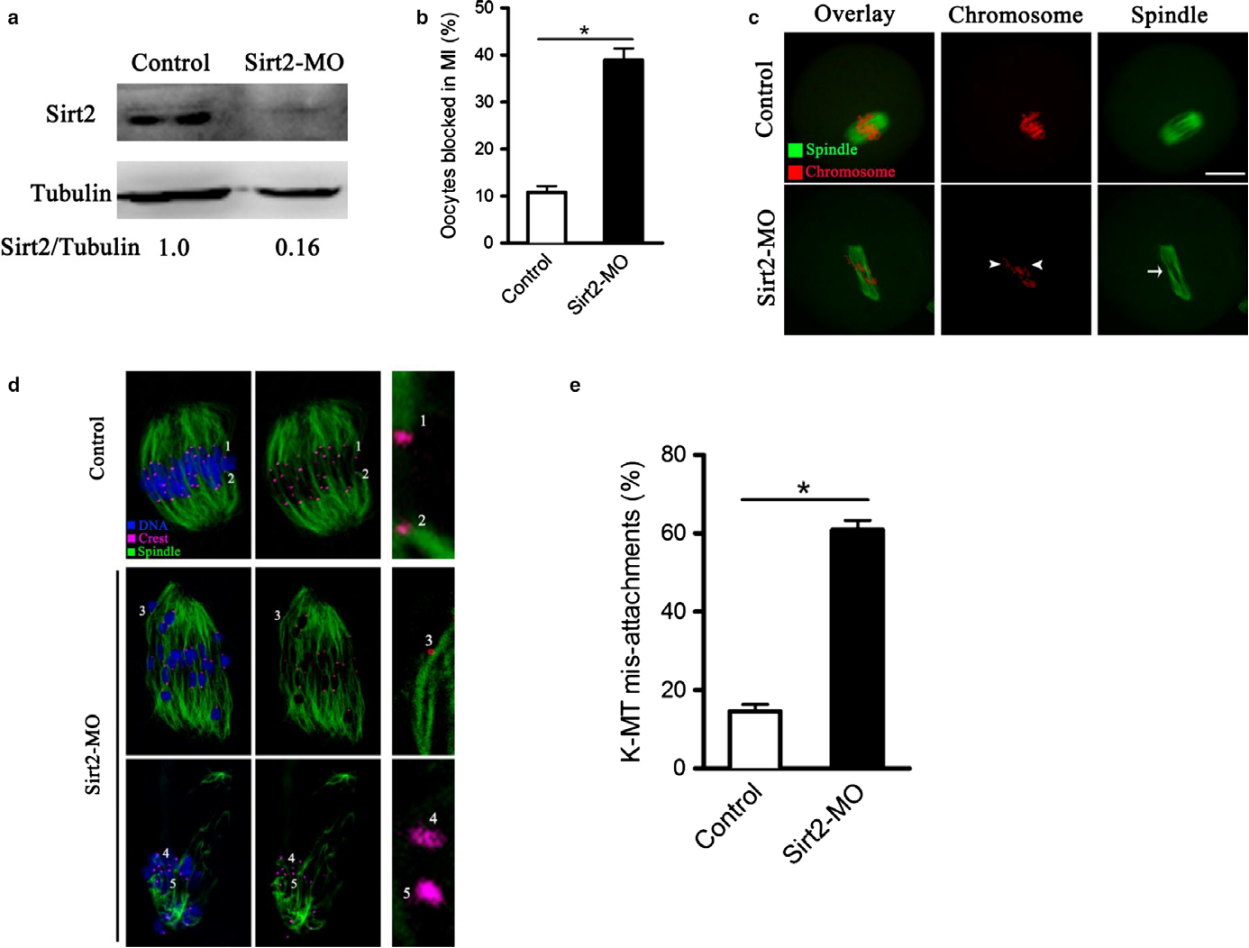
Effects of Sirt2 knockdown on maturational progression and meiotic structure in oocytes. Fully grown oocytes were injected with Sirt2‐MO and then cultured in vitro to evaluate the maturational progression and meiotic apparatus. (a) Western blotting showing the reduced expression of Sirt2 after MO injection. (*b*) Percentage of oocytes blocked in metaphase after Sirt2‐MO injection (*n* = 80 for control; *n* = 96 for Sirt2‐MO). (c) Control and Sirt2‐MO oocytes were stained with α‐tubulin antibody to visualize the spindle (green) and counterstained with PI to visualize chromosomes (red). Control metaphase oocytes present a typical barrel‐shape spindle and well‐aligned chromosomes. Spindle defects (arrow) and chromosome misalignment (arrowheads) were frequently observed in Sirt2‐MO oocytes. Representative confocal sections are shown. Scale bar, 25 μm. (d) Control and Sirt2‐MO oocytes at metaphase stage were labeled with α‐tubulin antibody to visualize spindle (green), CREST to detect kinetochore (purple), and co‐stained with Hoechst 33342 for chromosomes (blue). Representative confocal sections show the amphitelic attachment (chromosomes 1 and 2), and merotelic/lateral attachment (chromosome 3), and loss attachment (chromosomes 4 and 5) in Sirt2‐MO oocytes. (e) Quantification of control (*n* = 20) and Sirt2‐MO (*n* = 28) oocytes with K‐MT mis‐attachments. The graph shows the mean ± *SD* of results obtained in three independent experiments. *p < .05 vs. controls

To explore the function of Sirt2 in meiosis, fully grown GV oocytes were microinjected with specifically designed antisense morpholino of Sirt2 (Sirt2‐MO) to block the mRNA translation. The level of Sirt2 protein was efficiently knocked down evidenced by Western blotting analysis (Figure 1a). As we reported previously (Zhang et al., 2014), Sirt2 depletion had little effects on meiotic resumption, but markedly decreased the rate of Pb1 extrusion, indicating that Sirt2 is essential for maturational progression of mouse oocytes. To further define the developmental stage of those Sirt2‐MO oocytes without polar bodies, we performed nuclear staining and quantitative analysis. As shown in Figure 1b, approximately 35% of Sirt2‐MO oocytes were blocked in meiosis I, which was significantly higher than that of controls.

We also confirmed the effects of Sirt2 on meiotic apparatus in mouse oocytes. Anti‐α‐tubulin antibody was used to visualize the spindle (green) and chromosomes were stained with propidium iodide (PI; red). Consistent with our previous data (Zhang et al., 2014), the frequency of spindle defects and chromosome misalignment was dramatically elevated once Sirt2 was depleted (41.3% ± 2.1% vs. 10.7% ± 1.7% control; *p* < .05; Figure 1c). Chromosome segregation during meiosis requires a dynamic interaction between spindle microtubules and kinetochores, a macromolecular complex that localizes at the centromere of chromosomes (Mitchison & Salmon, 2001; Santaguida & Musacchio, 2009). The readily observed spindle/chromosome defects in Sirt2‐MO oocytes indicated that the kinetochore–microtubule (K‐MT) attachments are probably compromised. To evaluate K‐MT attachment status, we immunolabeled kinetochores with CREST and microtubules with antitubulin antibody as described previously (Ma et al., 2014). Amphitelic attachment (each kinetochore attached to one of the poles) is the predominant form in normal oocytes, as shown in Figure 1d (chromosomes 1 and 2). However, by performing quantitative analysis (Figure 1e), we found that the proportion of merotelic attachment (one kinetochore attached to both poles; Figure 1d, chromosome 3) and loss attachment (kinetochores unattached to either pole; Figure 1d, chromosomes 4 and 5) in Sirt2‐MO oocytes was significantly increased in comparison to control oocytes (60.9% ± 2.4% vs. 14.6% ± 1.8% control, *p* < .05). These K‐MT mis‐attachments would inevitably lead to unstable chromosome biorientation, which could contribute to the spindle/chromosome defects observed in Sirt2‐abated oocytes. Altogether, the results suggest that Sirt2 knockdown disrupts maturational progression and meiotic apparatus in mouse oocytes.

### 2.2 Acetylation of BubR1 at lysine 243 functions during mouse oocyte maturation

Lysine (K) 250 and 688 on BubR1 have been identified as the potential deacetylation targets of Sirt2 in human cells (North et al., 2014; Suematsu et al., 2014), which are corresponding to K243 and K657 residues in mice (Park et al., 2013).

**Figure 2:**
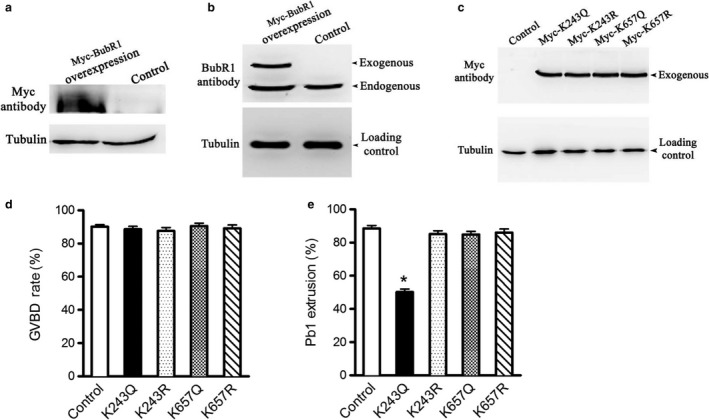
Effects of BubR1 acetylation on oocyte meiotic progression. Acetylation‐mimetic mutant BubR1‐K243Q/657Q or deacetylation‐mimetic mutant BubR1‐K243R/657R was microinjected into fully grown oocytes to evaluate the maturational progression. (a and b) Western blotting analysis showing that exogenous Myc‐BubR1 protein was efficiently overexpressed, probing with anti‐Myc and anti‐BubR1 antibody. Bands of endogenous and exogenous BubR1 protein are indicated. (c) Western blotting showing that the various mutant BubR1 proteins were expressed to the similar extent. (d and e) Quantitative analysis of GVBD and Pb1 extrusion rate in control and BubR1 mutant groups. Data are expressed as mean percentage ± *SD* from three independent experiments in which at least 100 oocytes were analyzed. *p < .05 vs. controls

To investigate whether BubR1 acetylation, and if so, which lysine residue(s) functions in oocyte meiosis, we constructed the site‐specific mutants (K‐to‐Q and K‐to‐R) targeting K243 and K657, and then, the mRNA encoding BubR1 mutant was microinjected into fully grown oocytes for phenotypic analysis. Substitution of lysine (K) with a glutamine (Q) mimics an acetylated amino acid state, while substitution with an arginine (R) mimics deacetylation (Choi et al., 2009). Immunoblotting verified that exogenous BubR1 protein was efficiently overexpressed in mouse oocytes (Figure 2a–b), and the various mutant BubR1 proteins were expressed to the similar extent (Figure 2c). After 3‐h release from meiotic arrest of GV oocytes in milrinone, control and all BubR1 mutant‐overexpressing oocytes resumed meiosis normally, indicated by similar GVBD rate (Figure 2d). However, oocytes expressing the K243Q mutant exhibited lower percentage of Pb1 extrusion at 14 hr compared to controls (Figure 2e), in line with the phenotypes of Sirt2‐MO oocytes. By contrast, none of K657Q, K657R, or K243R mutant showed an apparent effect on the meiotic progression. These data strongly suggest that acetylation status of BubR1‐K243 functions during oocyte meiosis.

### 2.3 Acetylation‐mimetic mutant BubR1‐K243Q impairs kinetochore–microtubule interaction in mouse oocytes

**Figure 3:**
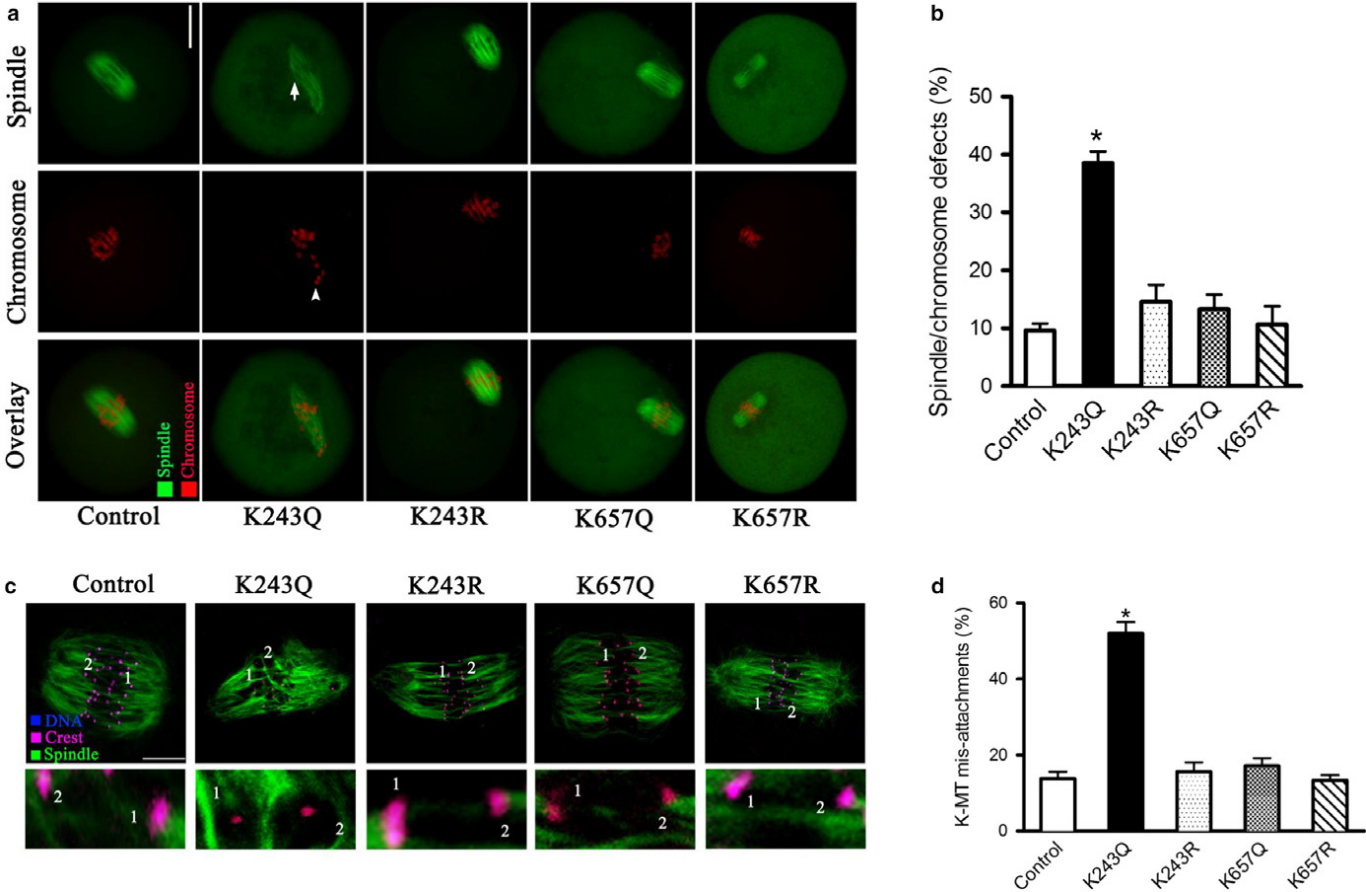
Effects of BubR1 acetylation on meiotic apparatus and kinetochore–microtubule attachment in mouse oocytes. (a) Control and BubR1 mutant‐injected oocytes were stained with α‐tubulin antibody to visualize spindle (green) and counterstained with PI to visualize chromosomes (red). Representative confocal sections are shown. (b) Quantification of control and BubR1 mutant‐injected oocytes with spindle/chromosome defects. Data are expressed as mean percentage ± *SD* from three independent experiments in which at least 100 oocytes were analyzed. (c) Control and BubR1 mutant‐injected oocytes at metaphase stage were labeled with α‐tubulin antibody to visualize spindle (green), CREST to detect kinetochore (purple), and co‐stained with Hoechst 33342 for chromosomes (blue). Representative confocal sections are shown. BubR1‐K243Q indicates the loss attachment between kinetochore and microtubule. (d) Quantitative analysis of K‐MT mis‐attachments in control and BubR1 mutant‐injected oocytes. Data are expressed as mean percentage ± *SD* from two independent experiments in which at least 20 oocytes were analyzed. *p < .05 vs. controls

To determine the role of BubR1 acetylation in the assembly of meiotic apparatus, mutant mRNA was injected into fully grown oocytes for spindle/chromosome analysis. Confocal microscopy revealed a significantly higher percentage of meiotic defects in BubR1‐K243Q oocytes relative to controls (33.2% ± 5.7% vs. 8.9% ± 2.8% control; *p* < .05; Figure 3a), showing diverse malformed spindles (arrow) and chromosome congression failures (arrowhead). These phenotypes differed sharply from metaphase spindles in control oocytes, which presented a typical barrel‐shape spindle and well‐aligned chromosomes. In contrast, ectopic expression of other mutants in oocytes had no evident effects on these meiotic structures (Figure 3a–b). Moreover, we detected a fourfold to fivefold increase in K‐MT mis‐attachments in oocytes expressing BubR1‐K243Q, while other mutants did not result in such deficits (Figure 3c–d). Collectively, these observations indicate that hyperacetylation of BubR1‐K243 disturbs the interaction between kinetochore and microtubule and thereupon causes the disorganization of meiotic spindle/chromosomes in oocytes.

### 2.4 Sirt2 promotes spindle assembly and chromosome alignment in oocytes through deacetylation of BubR1‐K243

**Figure 4:**
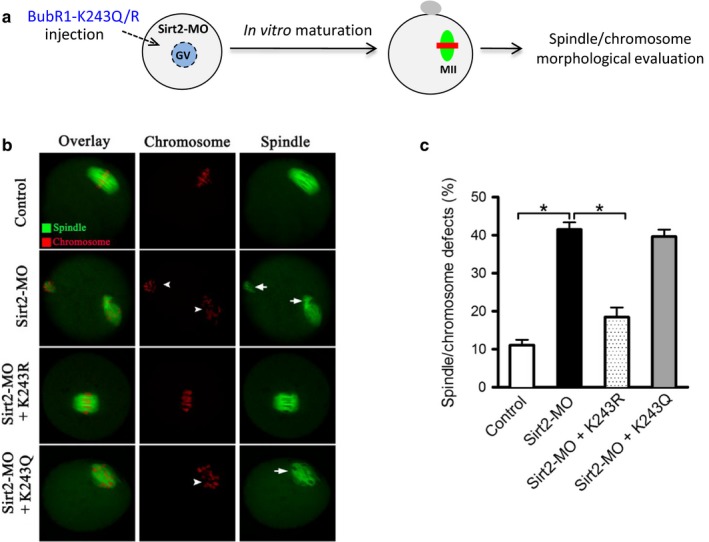
BubR1‐K243R partly rescues the meiotic defects in Sirt2‐depleted oocytes. (a) Schematic illustration of the experimental protocol to determine whether BubR1‐K243 acetylation mediates the effects of Sirt2 knockdown on oocyte phenotypes. (b) Control, Sirt2‐MO, Sirt2‐MO+BubR1‐K243R, and Sirt2‐MO+BubR1‐K243Q oocytes were stained with α‐tubulin antibody to visualize spindle (green) and counterstained with PI to visualize chromosomes (red). Representative confocal sections are shown. Spindle defects are indicated by arrows, and chromosome misalignments are indicated by arrowheads. (c) Quantification of control, Sirt2‐MO, Sirt2‐MO+BubR1‐K243R, and Sirt2‐MO+BubR1‐K243Q oocytes with spindle/chromosome defects. Data are expressed as mean percentage ± *SD* from three independent experiments in which at least 100 oocytes were analyzed. *p < .05 vs. controls

Given that Sirt2‐MO and BubR1K‐243Q oocytes show the similar phenotypes, and BubR1 was identified as a potential deacetylation target of Sirt2, we asked whether BubR1‐K243 acetylation mediates the effects of Sirt2 on oocyte meiosis. Toward this goal, a functional rescue experiment was conducted. Sirt2‐MO and BubR1‐K243 mutant mRNAs were simultaneously injected into oocytes, and then, meiotic phenotypes were examined (Figure 4a). As shown in Figure 4b–c, we found that the spindle defects and chromosome misalignment in Sirt2‐depleted oocytes were partially suppressed by the co‐expression of nonacetylated BubR1‐K243R mutant, whereas acetylated form BubR1‐K243Q could not. Therefore, these data indicate that lysine 243 is a major, if not unique, deacetylation site on BubR1α mediating the effects of Sirt2 on oocyte meiotic maturation.

### 2.5 Deacetylation‐mimetic mutant BubR1‐K243R ameliorates the defective phenotypes of oocytes from aged mice

Meiotic abnormalities and aneuploidy are much more prevalent in oocytes of women of advanced ages and are considered the major factors responsible for the increased incidence of infertility, miscarriage, and trisomic conceptions (Nagaoka et al., 2012). Although the consequences of aneuploidy in aged oocytes have been extensively studied, approaches to maintain fidelity of chromosome segregation during meiotic cell division are still elusive (Selesniemi, Lee, Muhlhauser, & Tilly, 2011). We previously showed that Sirt2 level is decreased in aged oocytes, and overexpression of Sirt2 reduces the penetrance of maternal age‐associated oocyte defects (Zhang et al., 2014).

**Figure 5:**
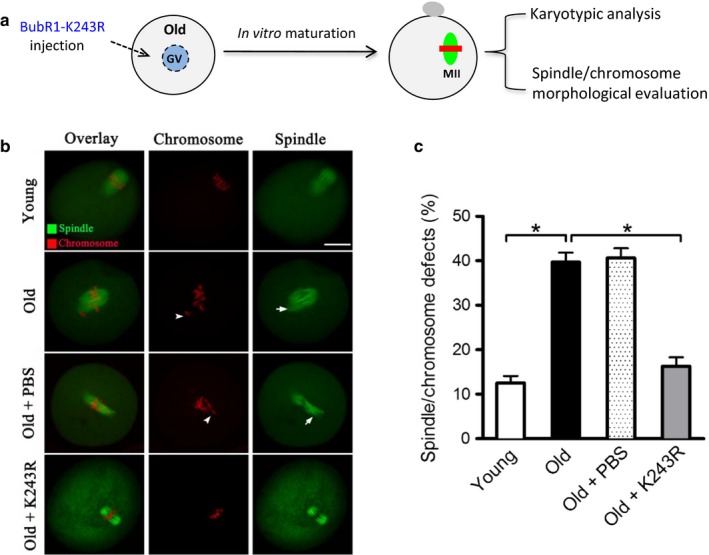
BubR1‐K243R overexpression ameliorates the maternal age‐associated meiotic defects in mouse oocytes. (a) Schematic illustration of the experimental protocol to determine whether BubR1‐K243 acetylation mediates the effects of maternal age on oocyte phenotypes. (b) Young, old, old+PBS, and old+BubR1‐K243R oocytes were stained with α‐tubulin antibody to visualize the spindle (green) and counterstained with PI to visualize chromosomes (red). Arrows indicate the chromosome misalignment, and arrowheads denote the spindle defects. Representative confocal sections are shown. (c) Quantification of young, old, old+PBS, and old+BubR1‐K243R oocytes with spindle/chromosome defects. Data are expressed as mean percentage ± *SD* from three independent experiments in which at least 120 oocytes were analyzed. Scale bar, 25 μm. *p < .05 vs. controls

As the involvement of BubR1 during oocyte aging has been widely reported (Baker et al., 2004; Riris et al., 2014), we next examined whether BubR1 mutant can rescue at least some of the phenotypic defects in oocytes from aged mice. For brevity, these oocytes are called “young oocytes” and “old oocytes” here. To this end, mRNA for BubR1‐K243R to mimic constitutively nonacetylated form was microinjected into fully grown old oocytes (Figure 5a), and PBS was injected as a control. Oocytes were in vitro matured and stained to check their spindle morphology and chromosome organization. Importantly, BubR1‐K243R significantly lowered the proportion of spindle/chromosome defects in old oocytes relative to controls (Figure 5b–c).

**Figure 6:**
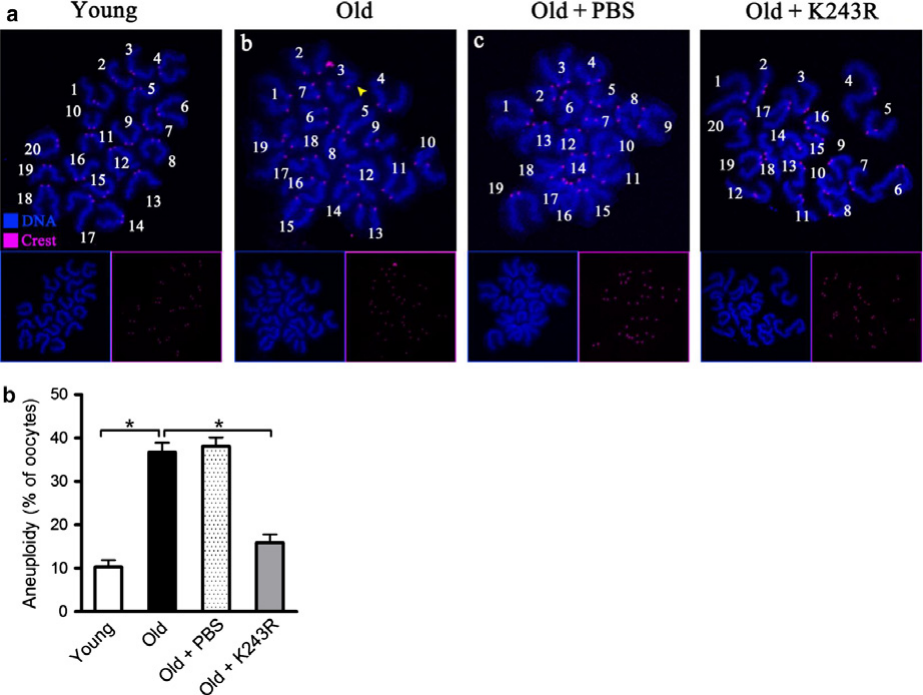
BubR1‐K243R overexpression reduces the aneuploidy incidence in oocytes from aged mice. (a) Chromosome spread of young, old, old+PBS, and old+BubR1‐K243R MII oocytes. Chromosomes were stained with Hoechst 33342 (blue), and kinetochores were labeled with CREST (purple). Representative confocal images showing the euploid young and old+BubR1‐K243R oocytes, and aneuploid old and old+PBS oocytes. (b) Histogram showing the incidence of aneuploidy in young (*n* = 32), old (*n* = 34), old+PBS (*n* = 40), and old+K243R (*n* = 42) oocytes. Data are expressed as mean percentage ± *SD* from three independent experiments. *p < .05 vs. controls

As these meiotic spindle defects in oocytes act to generate aneuploid eggs, we further evaluated the effects of BubR1‐K243R on the karyotype of matured oocytes by performing chromosome spreading combined with kinetochore labeling. As shown in Figure 6a–b, compared to young oocytes, about threefold increase in aneuploidy incidence was detected in old oocytes; however, forced expression of BubR1‐K243R mutant was able to markedly prevent the production of aneuploid eggs. Collectively, these results suggest that nonacetylated BubR1‐K243R is capable of protecting oocytes against maternal age‐associated meiotic defects.

## 3 DISCUSSION

Similar to mitotic cells (North & Verdin, 2007; North et al. 2003), we previously showed the enrichment of Sirt2 protein on the meiotic apparatus—the spindle and midbody during oocyte maturation (Zhang et al., 2014). In support of this observation, we here confirmed that Sirt2 knockdown in mouse oocytes results in spindle defects and chromosome disorganization, with impaired K‐MT attachment. Faithful chromosome separation is ensured by the bi‐oriented interaction of chromosomes to the spindle through the end‐on attachment of microtubules to kinetochores (Godek, Kabeche, & Compton, 2015). Hence, in Sirt2‐depleted oocytes, reduction in the K‐MT stability, which might, at least in part, contribute to the chromosome alignment failure observed in our experiments.

So far, several substrates of Sirt2 have been reported in different tissues and cell types mediating the diverse cellular processes. For example, histone H4K16 and α‐tubulin‐K40 have been indicated as the potential targets of Sirt2 to modulate chromatin conformation and microtubule stability (North et al., 2003; Vaquero et al., 2006). Sirt2 participates in myelin formation of Schwann cells by deacetylating partitioning defective 3 homologue (PAR3) (Beirowski et al., 2011). Sirt2 has roles in cellular response to glucose by deacetylating and stabilizing phosphoenolpyruvate carboxykinase 1 (PEPCK1), and thereby preventing its ubiquitylation‐dependent degradation (Jiang et al., 2011).

BubR1 is a component of SAC, a cell‐cycle surveillance system that prevents metaphase‐to‐anaphase transition by recruiting SAC proteins to this kinetochore and thus inhibiting the activation of APC/C (Jia, Kim, & Yu, 2013; Musacchio & Salmon, 2007; Sacristan & Kops, 2015). BubR1, as an integral part of the mitotic checkpoint complex (MCC), plays an essential role in this process. It has been demonstrated that BubR1 participates in the stabilization of K‐MT attachment by counteracting Aurora B phosphorylation through the recruitment of the B56 family of protein phosphatase 2A (PP2A) (Ditchfield et al., 2003; Kruse et al., 2013; Lampson & Kapoor, 2005; Varadkar, Abbasi, Takeda, Dyson, & McCright, 2017). Touati et al. recently showed that meiotic SAC was defective in BubR1 null oocytes and chromosomes were not aligned at the metaphase plate (Touati et al., 2015). Specifically, they found that cytoplasmic BubR1 is also required for the establishment of stable spindles in oocytes through an unknown direct or indirect pathway independent of its kinetochore localization. It is worth noting that these defective phenotypes of BubR1 null oocytes are very similar to what we observed in Sirt2‐knockdown oocytes (Figure 1).

BubR1 protein expression in natural aging mice is decreased in multiple tissues, including testis and ovary (Baker et al., 2004). High‐level expression of BubR1 extends mice lifespan and delays age‐related deterioration and aneuploidy (Baker et al., 2013). Similarly, a marked reduction of BubR1 levels was detected in oocytes from old women and aged mice (Lagirand‐Cantaloube et al., 2017; Pan, Ma, Zhu, & Schultz, 2008; Riris et al., 2014). Moreover, increasing evidence suggests that BubR1 insufficiency is closely associated with high frequency of spindle/chromosome defects and resultant aneuploidy in oocytes (Baker et al., 2004; Wei et al., 2010). Of note, BubR1 was identified to be acetylated at K250 at prometaphase in human cells. Chromosome segregation in cells expressing BubR1‐K250Q was delayed, whereas mitotic progression was accelerated in cells expressing BubR1‐K250R (Choi et al., 2009). Loss of BubR1 acetylation causes defects in SAC signaling and promotes tumor formation in mice (Park et al., 2013). Recently, North et al. revealed that acetylation at K668 promotes degradation of BubR1, and Sirt2 overexpression increases BubR1 protein levels by deacetylating BubR1 at this site (North et al., 2014). Here, we found that, in normal mouse oocytes, only BubR1‐K243Q mutant affects maturational progression and K‐MT interactions (Figues 2 and 3), while BubR1‐K243R is capable of preventing spindle abnormalities and chromosome misalignment in Sirt2‐MO oocytes (Figure 4). Due to the limitation of oocyte number and technical reason, we have not yet been able to directly dissect the relationship between BubR1 acetylation and Sirt2 activity in mouse oocytes. These findings support a concept that Sirt2‐dependent BubR1‐K243 deacetylation plays important roles in modulating the meiotic apparatus of oocytes. This study cannot and does not rule out that other substrates or pathways might be controlled by Sirt2 to influence meiosis in oocytes.

Importantly, we detected the marked reduction of Sirt2 in oocytes from aged mice (Zhang et al., 2014), and exogenous expression of BubR1‐K243R in these oocytes could alleviate the meiotic defects and aneuploidy generation (Figures 5 and 6). Altogether, our data identified Sirt2‐BubR1 as an important pathway determining the quality of aged oocytes, and we conclude that advanced maternal age induces the loss of Sirt2 protein in oocytes, which in turn leads to the hyperacetylation of BubR1‐K243, and, as a result, contributing to spindle defects, chromosome misalignments, and aneuploid eggs generation.

## 4 MATERIALS AND METHODS

All chemicals and culture media were purchased from Sigma (St. Louis, MO, USA) unless otherwise stated. Each experiment was repeated at least twice.

### 4.1 Mice

ICR mice were used in this study. To generate a natural aging mouse model, 42‐ to 45‐week‐old female mice which near the end of their reproductive lifespan were used. All experiments were approved by the Animal Care and Use Committee of Nanjing Medical University and were performed in accordance with institutional guidelines.

### 4.2 Antibodies

The following antibodies were purchased: rabbit polyclonal anti‐Sirt2 antibody (Cat#: AV32384; Sigma); mouse monoclonal FITC‐conjugated anti‐α‐tubulin antibody (Cat#: F2168; Sigma); mouse monoclonal anti‐Myc antibody (Cat#: ab18185; Abcam); human anticentromere CREST antibody (Cat#: 15‐234; Antibodies Incorporated); Cy5‐conjugated donkey anti‐human IgG (Cat#: 709‐605‐149 and 705‐095‐147; Jackson ImmunoResearch Laboratory).

### 4.3 Oocyte collection and culture

Female mice aged from 6 to 8 weeks or 42 to 45 weeks were used for collecting oocytes. To obtain fully grown GV oocytes, mice were superovulated with 5 IU pregnant mare serum gonadotropin (PMSG) by intraperitoneal injection. Cumulus enclosed oocytes were isolated 46–48 hr later, and denuded oocytes were released by repeatedly pipetting. Oocytes were cultured in drops of M16 medium covered with mineral oil at 37°C in the presence of 5% CO2. To obtain ovulated MII oocytes, female mice were superovulated by PMSG treatment followed by injection of human chorionic gonadotropin (hCG; 5 IU). Cumulus–oocyte complexes were isolated from oviducts, and cumulus cells were then removed by hyaluronidase incubation (1 mg/ml).

### 4.4 Plasmid construction and mRNA synthesis

Total RNA was extracted from mouse oocytes using Arcturus PicoPure RNA Isolation Kit (Applied Biosystems, Foster City, CA, USA). The primers used to amplify the CDS sequence of BubR1 and mutants are listed in Table S1 (Supporting information). PCR products were purified, digested with Fse I and Asc I (New England Biolabs, Beverly, MA, USA), then inserted into the pCS2^+^ vector with Myc tags. BubR1 mutants including K243Q, K243R, K657Q, and K657R were generated by site‐directed mutagenesis using pCS2^+^‐BubR1 as the template. K243Q/K657Q is a mutant version of BubR1 in which lysine 243/657 is substituted with glutamine, while such residue is replaced by arginine in K243R/K657R mutant. For mRNA synthesis, the plasmids were linearized by Not I, and in vitro transcription was conducted using mMessage mMachine T3 Kit (Ambion, Austin, TX, USA). Synthesized RNA was stored at −80°C.

### 4.5 Knockdown and overexpression analysis

Microinjection experiments were carried out using a Narishige microinjector. Morpholino (MO) or mRNA was injected to knock down Sirt2 or overexpress BubR1 mutants in mouse oocytes, respectively.

For knockdown experiments, morpholinos (MO) against Sirt2 were designed by Gene Tools (Philomath, OR, USA) through their site selection procedure, to target the initiation of translation. A nontargeting MO was injected as a control. MO was diluted with water to give a stock concentration of 1 mM, and then, 2.5 nl MO solution was injected into oocytes. For overexpression experiments, 10 pl BubR1 mutant mRNA solution (10 ng/l) was injected into the cytoplasm of GV oocytes. The same amount of RNase‐free PBS was injected as control.

Sirt2‐MO: 5′‐TCGGGACTGTCACCGACTGCTCTGT‐3′; CTRL‐MO: 5′‐CCTCTTACCTCAGTTACAATTTATA 3′.

After injections, oocytes were arrested at GV stage in medium containing 2.5 M milrinone for 20 hr to facilitate either mRNA degradation or translation and then cultured in milrinone‐free M16 medium for further experiments.

### 4.6 Western blotting

A pool of 150 oocytes was lysed in Laemmli sample buffer with protease inhibitor. Samples were separated on 10% SDS–polyacrylamide gels and transferred to PVDF membrane. Membranes were blocked with 5% nonfat dry milk in TBST (10 mM Tris, pH 7.5, 150 mM NaCl, and 0.1% Tween‐20) and then probed with primary antibodies overnight at 4°C (Sirt2 antibody, 1:800; Myc antibody, 1:1,000; α‐tubulin antibody, 1:2,000). After multiple washes in TBST and incubation with HRP‐conjugated secondary antibodies, the protein bands were visualized using an ECL Plus Western Blotting Detection System (GE Healthcare, Little Chalfont, UK).

### 4.7 Immunofluorescence

Immunofluorescence was performed as described previously (Wang, Chi, & Moley, 2012; Zhang et al., 2004). In brief, oocytes were fixed with 4% paraformaldehyde and permeabilized with 0.5% Triton X‐100 before blocking. Samples were incubated overnight at 4°C with FITC‐conjugated tubulin antibody to visualize spindle. To detect kinetochores, oocytes were co‐labeled with CREST (1:500). Chromosomes were evaluated by staining with propidium iodide (PI; 1:350) or Hoechst 33342 (1:300) for 10 min. After three washes in PBS, oocytes were mounted on antifade medium (Vectashield; Vector Laboratories, Burlingame, CA, USA) and then examined under a laser scanning confocal microscope (LSM 710; Carl Zeiss, Oberkochen, Germany).

### 4.8 Chromosome spread

Chromosome spread was conducted as described previously (Hunt et al., 2003). Briefly, MII oocytes were exposed to Tyrode's buffer (pH 2.5) for about 30 s at 37°C to remove zona pellucida. Samples (polar bodies had become detached and lost in most cases) were recovered in M2 medium for 10 min and then fixed in 1% paraformaldehyde with 0.15% Triton X‐100 on a glass slide. After air drying, oocytes were incubated with CREST overnight at 4°C and chromosomes were stained with Hoechst 33342. Samples were examined under a laser scanning confocal microscope.

### 4.9 Statistical analysis

Data are presented as means ± *SD*, unless otherwise state. Statistical comparisons were made with Student's *t*‐test and ANOVA when appropriate using Prism 5 software (GraphPad, San Diego, CA, USA). *p* < .05 was considered to be significant.

## CONFLICT OF INTEREST

None declared.

## AUTHOR CONTRIBUTIONS

DQ and QW designed research; XH, LH, XL, and JG performed research; DQ and QW analyzed data; DQ and QW wrote the article.
