# Supplementary material for: Sirt2‐BubR1 acetylation pathway mediates the effects of advanced maternal age on oocyte quality
Source: Aging Cell. 2017 Oct 25;17(1):e12698. doi: 10.1111/acel.12698 (PMC5770883; doi:10.1111/acel.12698)
Supplement: Supplementary file 1 [file ACEL-17-na-s001.doc]

**Supplementary Table 1** Primer sequences of genes for cDNA amplification

***Gene Primer sequence***

BubR1 Forward Primer: 5’ –GGGGGCCGGCCAGTTAGCACTCGGAGGATAAAGGTC – 3’

Reverse Primer: 5’ –GGGGGCGCGCCGACTGCTGATGTGTCTAAGTGTGCT– 3’

# Supplementary Table 2 Primer sequences of genes for site-directed mutagenesis of BubR1

***Gene Primer sequence***

BubR1 (K243Q) Forward Primer: 5’ –TCGGAGGTGCTCTGCAAGCTCCAGGTCAGA – 3’

Reverse Primer: 5’ –TCTGACCTGGAGCTTGCAGAGCACCTCCGA – 3’

BUBR1 (K243R) Forward Primer: 5’ –TCGGAGGTGCTCTGAGAGCTCCAGGTCAGA – 3’

Reverse Primer: 5’ –TCTGACCTGGAGCTCTCAGAGCACCTCCGA – 3’

BubR1 (K657Q) Forward Primer: 5’ –GGCCCTCATCATAAAGCAACTGAGCCCAATTATTG– 3’

Reverse Primer: 5’ –CAATAATTGGGCTCAGTTGCTTTATGATGAGGGCC – 3’

BubR1 (K657R) Forward Primer: 5’ –GGCCCTCATCATAAAGAGACTGAGCCCAATTATTG– 3’

Reverse Primer: 5’ –CAATAATTGGGCTCAGTCTCTTTATGATGAGGGCC– 3’
